# Could the Less-Than Subtotal Parathyroidectomy Be an Option for Treating Young Patients With Multiple Endocrine Neoplasia Type 1-Related Hyperparathyroidism?

**DOI:** 10.3389/fendo.2019.00123

**Published:** 2019-03-07

**Authors:** Fabio Luiz de Menezes Montenegro, Marilia D'Elboux Guimaraes Brescia, Delmar Muniz Lourenço, Sergio Samir Arap, Andre Fernandes d'Alessandro, Gilberto de Britto e Silva Filho, Sergio Pereira de Almeida Toledo

**Affiliations:** ^1^Parathyroid Unit- LIM-28, Laboratório de Cirurgia de Cabeça e Pescoço, Head and Neck Surgery, Department of Surgery, Hospital das Clinicas HCFMUSP, University of Sao Paulo School of Medicine, Faculdade de Medicina, Universidade de São Paulo, São Paulo, Brazil; ^2^Endocrine Genetics Unit (LIM-25), Endocrinology Division, Hospital das Clinicas, University of Sao Paulo School of Medicine, Faculdade de Medicina, Universidade de São Paulo, São Paulo, Brazil; ^3^Endocrine Oncology Division, Institute of Cancer of the State of Sao Paulo, University of Sao Paulo School of Medicine, Faculdade de Medicina, Universidade de São Paulo, São Paulo, Brazil

**Keywords:** parathyroidectomy, adolescent, multiple endocrine neoplasia type 1, hyperparathyroidism, hypoparathyroidism, surgery, parathormone, calcium

## Abstract

**Background:** The surgical treatment of primary hyperparathyroidism (HPT) in patients with multiple endocrine neoplasia type 1 (MEN1) has evolved due the concern of permanent hypoparathyroidism. As the diagnosis has increased, the extent of operation has decreased. Most MEN1 patients requiring parathyroidectomy are younger than 50 years and they pose a difficult balance to achieve between persistent HPT and life-long hypoparathyroidism. The aim of the present study is to review our experience with a large series of patients with MEN1-related HPT (HPT/MEN1) treated at a single institution in order to find clues to a better treatment decision in these younger cases.

**Method:** Retrospective analysis of consecutive HPT/MEN1 cases treated at a single institution with different operations: total parathyroidectomy and immediate forearm autograft (TPTX-AG), subtotal (STPTX), unintentional less than subtotal (U-LSTPTX) and intentional less than subtotal parathyroidectomy (I-LSTPTX).

**Results:** Considering 84 initial cases operated on since 2011 (TPTX-AG, 39; STPTX, 22, U-LSTPTX, 13, and I-LSTPTX, 10), the rates of hypoparathyroidism were 30.8% (U-LSTPTX), 28.2% (TPTX-AG), 13.6% (STPTX), and 0% (I-LSTPTX). Two-thirds of them (68%; 57/84) were young (<50 years) or asdolescents. MIBI scan was more sensitive to show parathyroid glands and bilateral disease. Considering the concordance of MIBI and ultrasound for the possibility of unilateral clearance, it would be suitable to 22.6% of the cases. Intra-operative parathormone showed a significant decay even after unilateral exploration, but longer follow up is necessary. Overall, there were seven (4%) adolescents in 161 cases treated from 1987 to 2018, three underwent TPTX-AG and four had U-LSTPTX. Five are euparathyroid, one had mild recurrence, and one required a reoperation after 8 years due to the residual gland.

**Conclusions:** Young patients are the most frequent candidates to parathyroidectomy. Less extensive procedures may be planned only if carefully reviewed preoperative imaging studies suggest a localized disease. Patients and their relatives should be fully informed of the risks and benefits during consent process. Future research with larger cohorts and long-term results are necessary to clarify if less than I-LSPTX or unilateral clearance are really adequate in selected groups of patients with HPT/MEN1 presenting lower volume of disease detected by preoperative imaging studies.

## Introduction

Multiple Endocrine Neoplasia Type 1 (MEN1) is an autosomal dominant syndrome with high penetrance and increased risk to a variety of benign and malignant neoplasms arising from both endocrine and non-endocrine tissues (1). The most common endocrine neoplasms are found in the pituitary, parathyroid glands, and in endocrine cells of the pancreas and duodenum. The prevalence of pituitary, enteropancreatic neuroendocrine, and parathyroid tumors are ~40, 57, and 95%, respectively (2). Affected individuals harbor germline mutations in the *MEN1* tumor suppressor gene, the first hit. The additional somatic *MEN1* mutation of the normal allele (the second hit) predicts coding of an inactivated MENIN protein, leading to tumor development (3). This mechanism may partially explain the MEN1 variable phenotypic expression, frequency, and why tumors are usually asynchronous in MEN1.

Parathyroid tumor in MEN1 is usually benign and functionally hyperactive, causing primary hyperparathyroidism (HPT). The clinical manifestations of MEN1-related HPT (HPT/MEN1) are rather similar to that of sporadic HPT cases, referring to bone loss and kidney stones. However, the onset of HPT/MEN1 usually occurs four decades earlier and its secondary bone and renal consequences are frequently more severe than in sporadic cases (4). Additionally, familial HPT is most often associated with multiglandular, asymmetric involvement of the parathyroid glands. Thus, parathyroid enlargement observed during parathyroidectomy (PTX) in HPT/MEN1 may involve from one to all glands. Parathyroid glands in HPT/MEN1 usually evolve to a neoplasm at different moments, depending on occasion that the second hit ensues in each gland. It may take a long time to other parathyroid glands to enlarge and some may never develop a tumor (5, 6).

Although the understanding of the disease pathophysiology and the drug development (such as calcimimetics) have evolved in the last years, the definitive treatment of HPT/MEN1 remains surgical. The indications for the operation follow those proposed for asymptomatic sporadic primary HPT, except for age (6). As the HPT/MEN1 may be identified early, deciding to operate only based on age would lead many young patients and children to surgery, with no clear evidence of health benefit. Besides, young patients may face frequent postsurgical complications, as recurrent laryngeal nerve damage and permanent hypoparathyroidism. However, many young patients do present early renal and/or bone disease requiring PTX (4).

The surgical treatment of HPT in MEN1 patients requires further technical skills to localize several possible affected/enlarged parathyroids, besides dissect the thymus in the upper mediastinum. However, hand dexterity is not enough. The surgeon must have also a sound judgment to manage normal and abnormal parathyroid tissue found during neck exploration (7).

The extent of parathyroid tissue resection in patients with MEN1-related HPT has evolved since the pre-genetic era. Before 1997 and routine genetic screening, the diagnosis of HPT/MEN1 was frequently performed in more advanced stages of the disease. As expected, the surgical findings were enlargement of all parathyroid glands, albeit they were frequently asymmetrical. Considering the limitations of imaging studies at that moment (with lower sensitivity and specificity), total parathyroidectomy, and immediate autograft (TPTX-AG) was often performed (8–10). However, the relatively high occurrence rate of permanent hypoparathyroidism, the observation of similar rates of recurrent disease in the long-run, the substantial improvement of parathyroid imaging studies, and the increase of the diagnosis of mild symptomatic or asymptomatic patients due to genetic screening led clinicians and surgeons to consider subtotal parathyroidectomy (STPTX) as the procedure of choice in HPT/MEN1 patients (11). Less than subtotal parathyroidectomy (LTSTPX) had only few proponents (12), probably due to the higher risk of recurrence and also the fear of complications that might result after a new surgical intervention on scar tissue of a previous bilateral cervical exploration.

The rationale of minimizing the operative risks (such as recurrent laryngeal nerve damage and permanent hypoparathyroidism), to facilitate a future neck exploration (not requiring dissection in postoperative fibrous tissue), to optimize the control of HPT, keeping these patients normocalcemic, and in compliance of thymus excision led some authors to propose a special type of LSTPTX in very selected cases. This surgery is performed in only one neck side, excising both ipsilateral parathyroids and thymus (9). This elegant proposal has as prerequisite the concordance in two or more preoperative imaging studies (scintigraphy, ultrasound, and four-dimensional computerized tomography) suggesting that there are only one or two enlarged glands at the same neck side. Despite short follow up, the results were promising and Versnick et al. (9) recommended it for young patients (9). The procedure has gained some acceptance, and it was later named as “unilateral clearance” (13). However, it is debatable and as other groups suggested high HPT recurrence rates favoring SPTX as the procedure of choice (14, 15). Children and young persons require a balance in the surgical excision to avoid the difficulties of managing permanent hypoparathyroidism (e.g., hypercalciuria, renal stones, cataracts, basal ganglia calcification) and the risks of neck reoperation (mainly due to possible damage to the recurrent laryngeal nerve).

In the present study we review our own experience with LSTPTX in MEN1 patients comparing it to cases submitted to classical surgical procedures as STPTX and TPTX-AG. Besides, we originally introduced the concept of “intentional” and “unintentional” LSTPTX and its utility to interpret surgical results more accurately. Also, by investigation of preoperative imaging studies, we estimated how many patients could be candidates to undergo less extensive procedures such as intentional LSTPTX and unilateral clearance.

## Materials and Methods

In our database 161 HPT/MEN1 patients underwent PTX at our tertiary academic hospital, Central Institute of the Hospital das Clinicas, from 1987 to 2018. Patients operated on before November, 2011 were previously reported in 2012 (16). In the present investigation, a retrospective analysis of patients submitted to PTX from 2011 onwards was performed. These patients are part of a MEN1 clinical and genetic screening program that has been performed since 1996 in our Service (1, 2, 4, 17–19).

### Criteria to Recommend PTX

Patients were referred to operation by the endocrinologist in charge of the case. Symptomatic patients were natural candidates to surgical treatment. Conversely, asymptomatic MEN-1 patients had a similar approach to the recommendations issued by the last Consensus Guidelines for Asymptomatic Sporadic Primary HPT for operation referral, except for age (20). Thus, asymptomatic patients with total calcium 1.0 mg/dL above the upper limit of the normality, osteoporosis or low bone mass for age, nephrolithiasis or nephrocalcinosis detected by imaging studies, reduced renal function (i.e., creatinine clearance < 60 ml/min/square meter) or 24 h calcium urinary excretion superior than 400 mg/dL were sent for operative treatment.

### Four Different Approaches to PTX

Due to its retrospective nature, some patients had a non-intentional LSTPTX, i.e., they were scheduled for STPTX or TPTX-AG, but less than three and half parathyroid glands were eventually excised (21). We grouped these cases under the denomination of unintentional LSTPTX (U-LSTPTX).

On the other hand, there were selected cases undergoing LSTPTX according to surgeon's discretion, and these were grouped as intentional LSTPTX (I-LSTPTX).

U-LSTPTX and I-LSTPTX groups are different at the start, although the final resection of the parathyroid tissue may be apparently similar. In the U-LSTPTX an extensive bilateral exploration was performed (possibly including central neck dissection, some sort of thyroidectomy, and thymectomy), as one or more glands were lacking. In contrast, in I-LSTPTX, the operation was mainly a unilateral approach or a limited bilateral approach, where normal glands were seen and preserved *in situ*.

STPTX was performed with bilateral exploration and the intent was to leave the amount equivalent to two normal parathyroid glands in a stump of the less diseased one or that of a more adequate gland in case of a possible future reoperation (trying to avoid a stump close to the recurrent laryngeal nerve). Bilateral thymectomy was routinely included.

TPTX-AG included the resection of all identified parathyroid tissue and immediate forearm autograft of 30 to 45 fragments of ~2 x 1 x 1 mm. Thymectomy was also routinely performed.

Possible complications secondary to PTX as nerve damage, hypoparathyroidism, persistent HPT, recurrence, and need for reoperation were included in the analysis.

### Intra-Operative PTH Measurements

At the institution, intra-operative PTH is included in the care of MEN1 patients. Currently, we routinely sample PTH five times during the operation. The first sample is at anesthesia induction from a peripheral vein (“peripheral baseline” sample) Then we switch the site of sampling to the internal jugular, vein right after its exposure in one side (“central baseline” sample). The following sample is obtained from the same initial point in the jugular vein, after the identification of all parathyroid tissue planned to be excised (“pre-excision” sample). After 10 and 15 min of the planned resection (TPTX-AG, STPTX, or LSTPTX) we sample PTH at the same internal jugular vein (samples “10 min” and “15 min, respectively). Additional samples are performed depending on the frozen section report or the results of previous samples. We considered a satisfactory decrease as the 80% reduction at the 10 min sample compared to the highest value obtained before in the jugular vein (either “central baseline” or “pre-excision”). The intraoperative decay of PTH was analyzed.

### Demography and Preoperative Biochemical Data

We reviewed demographic and preoperative data, including preoperative biochemical measurements of total calcium (Ca, normal range, 8.6–10.2 mg/dL), ionized calcium (Cai, 4.6–5.2 mg/dL), phosphorus (P, 2.7–4.5 mg/dL), and parathormone (PTH, 15–65 pg/mL).

### Age Groups

Attempting to better define which young patients could theoretically benefit from LSTPTX, we analyzed age at the surgery of all cases since 1987. According to the World Health Organization, all persons < 10 years old are children, and adolescents are individuals aged 10–19 years inclusive (22). The Consensus on Asymptomatic Hyperparathyroidism defines young patients as younger than 50 years (20). Thus, we stratified these groups as follows: children < 10 years, adolescents those between 10 to 19 years and young adult patients those aged 20 to 50 years.

### Imaging Studies and Unilateral Clearance

We reviewed the results of preoperative imaging with ^99m^Tc-sestamibi (MIBI) scintigraphy and neck ultrasound imaging (US). We checked the results of MIBI scan and neck US as the possible guiders of I-LSTPTX. We considered only the results of the MIBI scan or US performed at the institution. Some patients had these localizing studies from other facilities before the operation and they were not considered for the present evaluation. MIBI scan was usually done by planar images 15 min, 2 h, and 5 h after injection. At discretion of the Nuclear Medicine, some patients had MIBI-SPECT and very few had a MIBI-SPECT-CT in the current analysis. We considered the report of the specialist to count on positivity or negative, the number of parathyroids identified, and laterality. US were always performed at the radiology department by different ultrasonographers and different machines. Exactly as for the MIBI, the report of the specialist was considered for the same items.

To estimate how many patients would theoretically be suitable to undergo an I-LSTPTX we considered the recommendation that only one gland or two ipsilateral glands were evident on each examination separately and in combination. If both results were negative, I-LSTPTX was considered not suitable as bilateral exploration was deemed as the procedure of choice. However, if one of the imaging studies was negative and the other indicated only one enlarged gland, then we considered this combination as feasible for unilateral clearance. We studied the number of glands suggested by each method and the frequency of these findings in the different age groups.

### Additional Cases of I-LSTPTX Operated at Other Hospitals

We added three cases of I-LSTPTX that were operated on by us at other facilities, as the standard of operative care was rather similar.

### Statistical Analysis

Continuous variables were tested for normality using the Kolmogorov-Smirnov test and were represented by mean and standard deviation (SD) when parametric, or by median, and inter-quartile range (Q1-Q3) when non-parametric. Nominal data are presented by frequency. For statistical inferences of continuous variables, Student‘s the test or non-parametric tests were employed accordingly. For counts, inference was analyzed by Qui-square or Fisher exact test, when adequate.

This study is part of a project approved by the Institutional Review Board and it is supported by grants of FAPESP (2016/25594-9; 2016/07504-2; 2015/25444-4).

## Results

### Age at the Operation

From 2011 to August, 2018, there were 98 operations in 94 MEN1 patients. We had five cases with previous operations at other facilities and referred for remedial surgery. There were nine reoperations related to our own previous cases. Four of these were patients with their initial operation during 2011–2018 and five were operated on before 2011. Details of these reoperated cases are presented below. Mean age was 43.5 years (range, 14–78 y). There were 58 females (mean age 45.3 years) and 35 males (mean age 40.6 years). No statistically significant difference occurred in age and sex distribution comparing present cohort with our past experience (1987–2011) (16).

**Figure 1:**
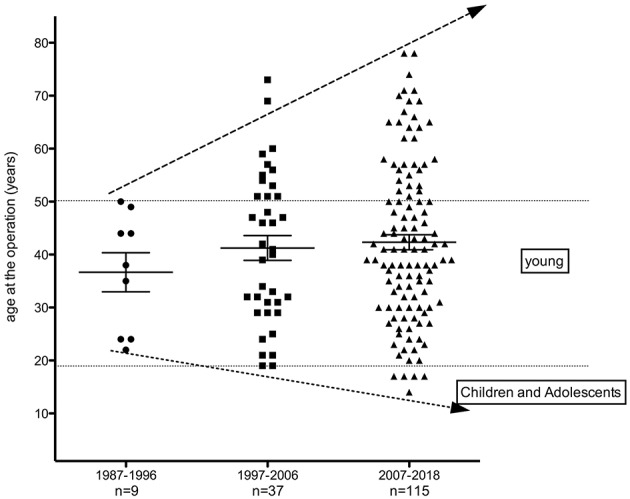
Scatter plot of age according to the vintage of treatment from patients with MEN1-related hyperparathyroidism undergoing parathyroidectomy (1987–2018).

Noteworthy, the number of HPT/MEN1 cases has substantially increased in the last two decades, coinciding with the available routine genetic analysis of *MEN1* gene. The scatter plot of age distribution at the surgery in three decades is shown in Figure 1.

Seven patients (4%) in the second decade of life (≤ 21 y) and no case in the first decade (≤ 10 y) with MEN1-related HPT were submitted to PTX in our group. None of them (0/9; 0%) underwent PTX during 1987–1997; two (2/37; 5.4%) were operated on during 1997–2006; and five (5/115; 4.3%) during 2007–2018. Thus, proportion of patients under 20 y of age has significantly increased from 0% in 1987–1996 to 4.3% in 2007–2018 (*p* < 0.05). Although the number of patients younger than 21 y in our cohort is limited, 75% of the patients are younger than 50 years of age. Similarly, the routine search for *MEN1* mutations in our institution after 1997 led to substantial increase of genetic carriers and clinical recognition of MEN1-related HPT cases between 20 and 50 years. Thus, cases with 20 −50 y rose from 9 to 23, respectively, in 1987–1996 and 1997–2006, to 75 in 2007–2018. In addition, an increasing number of HPT/MEN1 cases were operated on, as 9, 37, and 115 patients were, respectively, submitted to PTX in 1987–1996, 1997–2006, and 2007–2018 time intervals (Figure 1).

### Reoperations

Of the 98 PTX between 2011 and 2018, 14 of them were reoperations: five patients underwent previous neck procedures at other hospitals, ranging from single gland resection to a previous TPTX-AG, with a residual supernumerary gland. Of our own nine reoperated cases, three had residual neck parathyroids glands, two had graft dependent recurrences, two underwent cryopreserved parathyroid autografts; one had a supernumerary parathyroid gland; and one was operated on due to HPT persistence after a STPTX. An irrefutable pathogenic germline variant was identified in *MEN1* gene in 71 of these cases operated between 2011 and 2018 by Sanger sequencing or multiplex ligation-dependent probe amplification (1, 2, 4, 10, 17, 19).

### MIBI Scintigraphy and Neck US

We could retrieve information of MIBI scan in 71 patients and neck ultrasound in 64 patients. Table 1 presents the number of glands detected by MIBI and US. Table 2 shows the lateralization in detected PT glands, suggested by MIBI/US imaging studies.

**Table 1:** Number of abnormally enlarged parathyroid glands suggested, respectively by MIBI and US in 71 and 64 HPT/MEN1 patients.

**Number of glands**	**Zero**	**One**	**Two**	**Three**	**Four**	**Five or more**
MIBI *n* = 71	6 (8.4%)	22 (31.0%)	22 (31.0%)	14 (19.7%)	7 (9.9%)	0 (0.0%)
Ultrasound *n* = 64	17 (26.6%)	27 (42.2%)	13 (20.3%)	2 (3.1%)	5 (7.8%)	0 (0.0%)

**Table 2:** Laterality suggested by preoperative imaging studies using both MIBI and US in 71 and 64 HPT/MEN1 patients, respectively.

	**Negative result**	**Unilateral**	**Bilateral**
MIBI	6 (8.4%)	23 (32.4%)	42 (59.2%)
Ultrasound	17 (26.6%)	28 (43.8%)	19 (39.7%)

MIBI had a significantly lower frequency of negative results, as compared to US (*p* = 0.006, Fisher's exact test). Conversely, MIBI was more frequently associated with positivity in more than one gland, as compared to US (*p* = 0.02, Fisher's exact test).

MIBI had a higher ability to show bilateral disease (*p* = 0.01, 59.2% vs. 39.7%; Fisher's exact test).

The mean age of patients with unilateral report in MIBI was 37.6 y (SD 11.5), and 39.0 y (SD 13.7) for US. The mean age for bilateral reports in MIBI was 41.9 y (SD 15.1) and 39.3 y (SD 12.8) in US. Mean age of the cases with a negative MIBI was 54.0 y (SD 19.2) and 48.8 y (SD 19.2) for US. Although there was an apparent tendency to negative results in older individuals and unilateral disease in the group operated in younger age, it was not statistically significant (1-way ANOVA test *p* = 0.05 for MIBI, and *p* = 0.08 for US).

Both MIBI and US reports were available in 62 cases. Table 3 shows the concordance and discordance in their results, and the mean age of these patients in each situation.

**Table 3:** MIBI and Ultrasound concordance or discordance.

**MIBI + Ultrasound**	**n (%)**	**Age (years) Median (Q1-Q3)** **(Range)**
Concordant Negative	3 (4.8%)	55, 69, and 78^*^
Discordant	33 (53.2%)	38 (24–52) (17–71)
Concordant Unilateral	14 (22.6%)	37 (35–40) (14–66)
Concordant Bilateral	12 (19.4%)	40 (31–45) (17–71)
Total	62 (100%)	38 (30–50) (14–78)

* *n too small, absolute values are presented*.

Discordance was most observed when there was a bilateral positive MIBI scan and unilateral positive US imaging. This finding was detected in 13 patients (21%). Other cases of discordant results were as follows: MIBI positive bilaterally and US negative seen in 10 cases (16%); MIBI positive unilaterally and US bilaterally in 4 cases (6.4%); MIBI positive unilaterally and US negative in three cases (4.8%); MIBI negative and US unilateral in two cases (3.2%) and MIBI negative and US bilaterally in one case (1.6%).

There was no significant difference as to age regarding the concordance or discordance in imaging studies.

### Type of Operation and Follow Up

Excluding reoperations, we had 84 initial cases to evaluate since 2011. Median preoperative values (interquartile range) were as follows: Ca = 10.9 mg/dL (10.4–11.4), Cai = 6.0 mg/dL (5.7–6.3), *P* = 2.6 mg/dL (2.3–3.1) and PTH 136 pg/mL (101–202). Two-thirds of them (68%; 57/84) were HPT cases operated in young age: 54 (20–49 y) and three adolescents (14–17 y). One quarter of cases were younger than 30 y. Overall, we had one laryngeal recurrent damage (1.2%). This patient had a metastasis of a thoracic carcinoid to the right superior parathyroid, possibly contributing to a difficult nerve dissection.

Table 4 shows the number of patients undergoing each procedure and their characteristics. Although the hypoparathyroidism rates of 28.2% (TPTX-AG) and 30.8% (U-LSTPTX) are apparently higher than the rate of 13.6% of STPTX, there was no statistical significance (qui-square *p* = 0.33).

**Table 4:** Clinical data, biochemical parameters, and outcome of HPT/MEN1 patients according to the surgical procedure.

	**TPTX-AG**	**STPTX**	**I-LSTPTX**	**U-LSTPTX**
*n*	39	22	10	13
Age (SD)	42 y (14)	43 y (16)	50 y (17)	42 y (18)
F:M	25:14	13:9	7:3	8:5
**OPERATION VINTAGE**				
2011–2014	21	0	1	11
2015–2018	18	22	9	2
% intraoperative	88%	84%	88%	90%
PTH decrease (10min) (SD)	(78–95)	(69–88)	(79–84)	(82–96)
**Median PTH**				
Pre-surgery	179	104	123	155
6 months after surgery	18	38	49	21
CaT (pre-surgery)	11.1	10.7	10.8	10.2
CaT (6 months after surgery)	9.0	9.3	9.6	9.5
Cai (pre-surgery)	6.1	5.9	5.8	5.5
Cai (6 months after surgery)	4.8	5.0	5.0	4.4
P (pre-surgery)	2.6	2.4	2.6	2.7
P (6 months after surgery)	3.8	3.2	3.2	3.9
**Outcome n (%)**				
Hypoparatyroidism	11 (28.2%)	3 (13.6%)	0 (0.0%)	4 (30.8%)
Euparathyroidism	19 (48.7%)	14 (63.3%)	4 (40.0%)	6 (46.2%)
Persistent HPT	2 (5.1%)	3 (13.6%)	1 (10.0%)^*^	1 (7.7%)
Recurrent HPT	2 (5.1%)	1 (4.5%)	0 (0.0%)	1 (7.7%)
Not available^**^	5 (12.8%)	1 (4.5%)	5 (50.0%)	1 (7.7%)

TPTX-AG, total parathyroidectomy and immediate autograft; STPTX, subtotal parathyroidectomy (PTX); I-LSTPTX, intentional less than subtotal PTX; U-LSTPTX, unintentional less than subtotal PTX/

* case considered as persistence due to ionized calcium of 5.59 mg/dl and PTH 57 pg/mL at 6 months, but patient is still under follow up to a final diagnosis./

** *Lost to or too short follow up*.

**Figure 2:**
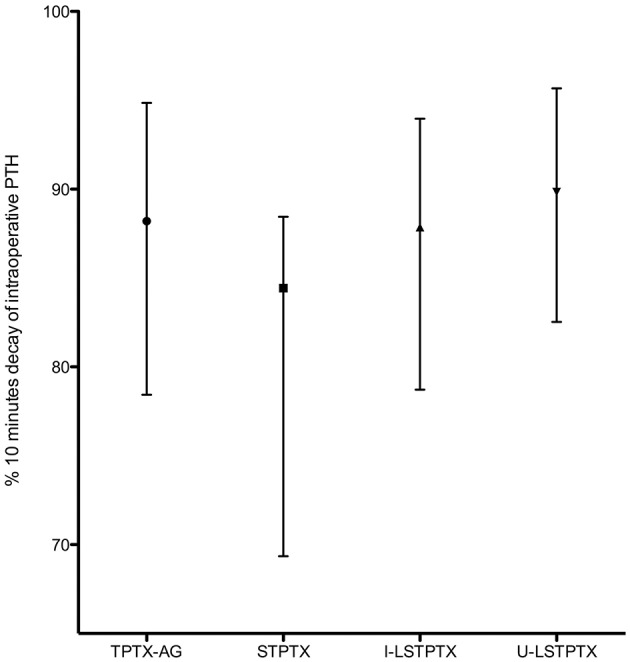
Median and interquartile range of the intensity of intraoperative PTH decay at 10 min.

The intraoperative PTH values showed very similar decay profile irrespective to the type of operation, as shown in Figure 2. The intensity of decay is apparently less intense in the STPTX, but with no statistical significance.

There was no correlation with the intensity of intraoperative PTH drop and the long-term outcome. Within six cases with persistent HPT, three showed an intraoperative PTH drop below 75%, comparing with intraoperative basal values. There was a significant proportion of euparathyroid cases (43 patients). In most of them (38 cases), the PTH values dropped more than 75% 10 min after parathyroid resection (*p* = 0.047, Fisher's exact test) while only five of them had a decrease lower than 75%. Interestingly, all but one patient undergoing I-LSTPTX presented reductions of 75% or more in PTH values at 10 min after the planned resection. In two cases, the decay was between 75 and 79%. In the remaining seven cases, PTH levels reduced between 82 and 99% comparing to baseline measurements. The seven latter cases, submitted to unilateral clearance at institution, had a mean decrease of intra-operative PTH values of 83% (SD 12%).

**Figure 3:**
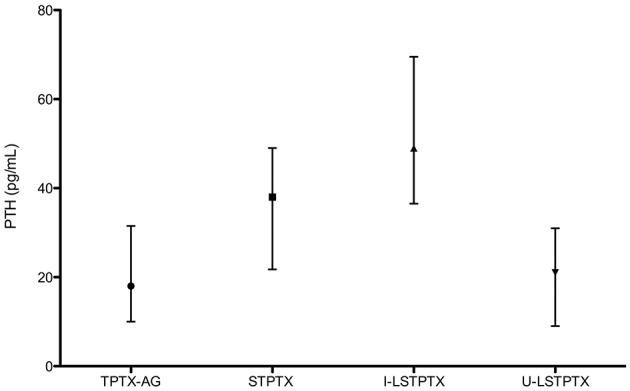
Median and Interquartile range of PTH 6 months after surgery (according to the type of operation).

Figure 3 shows PTH levels 6 months after surgery in each four different groups. There were significant differences between TPTX-AG when compared to STPTX (*p* = 0.004) or I-LSTPTX (*p* = 0.002). I-LSTPTX also differed from U-LSTPTX (*p* = 0.002). The difference of U-LSTPTX and STPTX had a *p*-value of 0.04. Of note, PTH after TPTX-AG and U-LSTPTX had no difference (*p* = 0.62). Levels after STPTX and I-LSTPTX were not statistically different (*p* = 0.12).

### Unilateral Clearance

Three patients submitted to unilateral clearance operated by the authors at other hospitals were added to the analysis. Altogether we have ten cases, seven were females. In eight cases, clearance was on the left side. Some of their individual data are presented at Table 5.

**Table 5:** Patients with HPT/MEN1 submitted to unilateral clearance.

**Case**	Age^*^	**Mutation positive**	**Outcome** **follow up**	**Intra-operative** **PTH decay (%)**	**Preoperative** **CaT (mg/dL)**	**Next day** **CaT (mg/dL)**	**Preoperative** **PTH (pg/mL)**	**Next day** **PTH (pg/mL)**	**Next day** **PTH decay (%)**
1	Old	No	Unknown^**^	93.6	10.7	8.7	207	22	89.4
2	Old	Yes	Unknown^**^	75.1	10.4	9.1	59	24	59.3
3	Young	No	Euparathyroid 12 months	86.8	12.6	9.4	1455	9	99.4
4	Young	Yes	Unknown^***^	79.9	9.6	8.7	86	27	68.6
5	Young	Yes	Unknown^***^	60.4	9.9	9.5	74	30	59.5
6	Young	No	Euparathyroid 13 months	82.6	11.4	9.3	129	10	92.2
7	Old	Not available	Mild persistance 6 months	94.9	11.8	8.2	228	35	81.0
8	Old	No	Unknown^**^	77.7	10.1	8.7	130	10	92.3
9	Young	Yes	Unknown^***^	89.8	9.2	8.5	373	33	91.2
10	Young	Yes	Euparathyroid 12 months	72.1	12.0	9.8	78.2	0	100.0

* Age: young (20–50 years), old (>50 years)

** *-lost to follow up*,

*** *- < 4 months*.

### Adolescents

The first adolescent was treated at 2002 with a U-LSTPTX. Presently, he has mild elevated calcium, but he has not fulfilled criteria for reoperation 16 years after first surgery. Table 6 includes data of all the seven adolescent patients.

**Table 6:** Experience with seven adolescent patients with MEN1-related HPT submitted to parathyroidectomy.

**Case**	**Year**	**Type of operation**	**Outcome (from operation until the present)**
1	2002	U-LSTPTX	Mild recurrence, no reoperation required
2	2006	U-LSTPTX	Euparathyroid
3	2008	U-LSTPTX	Recurrence, reoperation due residual gland after 8 years
4	2009	TPTX-AG	Euparathyroid
5	2014	U-LSTPTX	Euparathyroid
6	2017	TPTX-AG	Euparathyroid
7	2018	TPTX-AG	Euparathyroid

*U-LSTPTX, unintentional less than subtotal parathyroidectomy; TPTX-AG, total parathyroidectomy followed by immediate forearm auto-graft*.

## Discussion

The present results underline the urgent need to further discuss on the best surgical protocol in patients with MEN1-related HPT. Overall, surgeons choose STPTX or TPTX-AG as pattern surgical procedure in their services independent of previous images studies or age (23, 24). We postulated that the extent of parathyroid tissue resection in patients with MEN1-related HPT could be influenced for age-dependent factors and for lower volume of disease detected by preoperative imaging studies. By less extensive surgeries, it would be possible minimize, avoid or postpone long-standing permanent hypoparathyroidism, mainly in the subset of young cases (< 50 y) that represent the age group more commonly underwent to surgical treatment in MEN1.

Classically, STPTX and TPTX-AG have equivalent rates of persistent and recurrent HPT (25). Overall, most services have preferred STPTX by lesser rate of permanent hypoparathyroidism. In fact, the rate of hypoparathyroidism varies in different series from 0 to 50% after TPTX-AG and 0–35% after STPTX (25). However, the prevalence of permanent hypoparathyroidism after STPTX is not irrelevant and it may be aggravated as most MEN1 patients are diagnosed and operated for HPT in young age (< 50 y). In this young subset, chronic complications associated with long-term permanent hypoparathyroidism represent a real risk, but they have not been reported so far. A worst scenario may be anticipated if permanent hypoparathyroidism develops during infancy and adolescence after extensive surgical procedures.

So far, limited data are available on PTX in very young patients (< 21 y) with HPT/MEN1, mostly related with the best surgical approach this age group (26, 27). Besides, the number of HPT/MEN1 cases submitted to PTX during infancy and adolescence before the advent of routine clinical and genetic MEN1 screening was very limited, as diagnosis, and surgical treatment mostly occurred after ages of 21 y (5). After routine MEN1 screening much more adolescent MEN1 cases have been diagnosed with HPT fulfilling criteria for PTX (26–28). Concordantly, between 1987 and 1996 we had no adolescent patient submitted to PTX, whereas since 1997 the number of cases aged < 20 y has increased substantially, achieving 4% of our cohort. Remarkably, 25% of our cases are now younger than 30 y. The absence of children with HPT/MEN1 in our sample is not unexpected, as cases ≤ 11 y are exceedingly rare even in large multi-institutional series. In a series of 160 cases younger than 21 years, Goudet et al. (26) reported that only one patient underwent PTX before the age of 10 years (26).

Contrasting with a high penetrance of HPT/MEN1 at 20 y (50–60%), PTX has been conducted in a few MEN1 adolescents (27–29) probably because cases are predominantly asymptomatic and the ideal timing to indicate surgery for HPT/MEN1 remains undefined being more debatable in this age group yet (29) As a specific guideline for very young MEN1 cases are still lacking, the indication for PTX in affected adolescents should take in account several aspects that are characteristic of this age group, as be cautious with and to avoid the possible prejudice of bone accretion and peak of bone mass in early life. In addition, hypothyroidism should be avoided to allow normal growth (6).

Based on this scenario, less extensive surgical protocols could be an alternative to avoid or minimize risks of chronic complications in adolescents or same in young cases (< 50 y) operated by HPT/MEN1. To approach this issue, we examined the performance of different PTX options that were conducted in patients with HPT/MEN1 of different age groups and that could be adapted to adolescent patients, even though this approach is speculative. Indeed, our cohort included groups of HPT/MEN1 patients underwent to different surgical procedures as TPTX-AG, STPTX, and LSTPTX operated in single institution allowing comparisons. These comparisons are amplified as we originally introduced the concept of intentional and non-intentional LSTPTX. Besides, this series allowed additional comparisons with a group of HPT/MEN1 cases underwent to unilateral clearance, a special type of LSTPTX. From our knowledge, this is the first study comparing all these surgical procedures conducted in a single institution.

Although a longer follow up and a higher number of cases in different ages underwent to I-LSPTX sustained by pre-operative image studies is needed, the present data appoint that in selected HPT/MEN1 cases presenting with mild HPT disease and apparently lateralized parathyroid disease by preoperative imaging, I-LSTPTX may represent an option to more extensive procedures in the subset of young MEN1 patients. It is possible that children and adolescents represent the group with higher potential benefits with this strategy. In this specific subset of cases, HPT may be adequately controlled, minimizing the risk of hypoparathyroidism, as seen in our series including mainly young cases (< 50 y) operated. In our experience, patients undergoing I-LSTPTX and also in those submitted to unilateral clearance were not adolescent patients. However, even if recurrence would be inevitable, it may take several years until a second intervention is required. Meanwhile, unilateral clearance approach in selected cases may give a chance of long-term normal bone modeling allowing to reach the maximum peak of bone mass until recurrence ensues.

Of note, in our experience, even STPTX had a frequency of hypoparathyroidism of almost 14%, which may have an inadequate impact on bone formation of adolescent or same of patients younger than 30 y (25% of our casuistic). Goudet et al. (26) reported 11% of hypocalcemia in 37 young patients submitted to either STPTX or LSTPTX. Interestingly, this low rate of hypocalcemia could be partially explained by the predominance of LSTPTX (25 cases, 68%) in their cohort. Despite marked predominance of LSTPTX, they reported that 67% of cases were normocalcemic at the last follow-up before 21 year old and only two were re-operated before the age 21 y (26). These data reinforce our hypothesis that the selection for less extensive procedures in adolescents should be deeply investigated.

Curiously, we had similar rate of hypocalcemia in our group U-LSTPTX compared to STPTX and TPTX-AG, indicating that extensive exploration of cases with normal parathyroids trying to achieve the planned STPTX or TPTX-AG may amplify the risk of hypoparathyroidism. Indeed, the rate of hypoparathyroidism in U-LSTPTX was the highest. As expected, we had no hypocalcemia when unilateral clearance or I-LSTPTX was supported.

More recently, Vannucci et al. (27) reported six MEN1 adolescents with HPT and control of calcemia for different therapeutic strategies varying from calcimimetics to TPTX-AG. In a subsequent report of the same group with 14 cases diagnosed during second decade of life and operated for HPT before 25 years, most cases had TPTX-AG (eight cases) and two of them were hypocalcemic in last follow-up. Conversely, none of their four cases submitted to less extensive procedures (partial parathyroidectomy) had hypocalcemia (29). However, in half of this latter group, a second partial surgery was performed due recurrent HPT from three up to 14 years after first surgery and in one they performed a TPTX-AG after persistent disease. They had only one recurrence in autograft in case who underwent TPTX-AG (29). In our study, within four young cases who underwent to U-LSTPTX, two remain normocalcemic four and 14 years after surgery and other two had recurrence after 8 years, one of them needing of second surgical procedure while other follows with mild HPT 16 years after PTX.

As unilateral clearance is based on the assumption of presence of unilateral HPT disease at that specific moment, we can estimate that the decision based only in preoperative US would allow LSTPTX in almost 44% of the cases, while if based solely on MIBI scan it would be indicated to 32% of patients. Also, if for surgical decision one considers both US and MIBI results, it would be reasonable to indicate LSTPTX in 23% (concordant unilateral) and possibly to another 8% more, if we accept one unilateral positive imaging and the other negative. As MIBI is more capable to detect multiglandular disease, it seems advisable to routinely perform MIBI scan in the preoperative period of PTX, if one is considering an I-LSTPTX for a selected patient.

In addition, four-dimensional computerized tomography is gaining wide acceptance as a very sensible method (30). We still have very limited experience with four-dimensional tomography in HPT, and more notably in MEN1 patient. Its indication for adolescents with MEN1-related HPT seems to be limited as one of its major drawbacks is the intensity of radiation exposure and the risk of future development of neoplasms in the surrounding tissues (31).

In patients with one single parathyroid gland evidenced in MIBI scan, a I-LSTPTX could be discussed with them and/or parents, stressing that there is some expectation of HPT control, especially if intraoperative PTH is available, without imposing difficulties for a future operation should this need arise. So far, in our own cases only one adolescent required reoperation and it was necessary 8 years after U-LSPTX.

Intraoperative PTH should be used with caution in MEN1 patients, as it usually fails if the 50% criterion is employed (32). A higher cutoff as 75% seems to be more adequate in these patients, but even drop rates higher than 90% may be associated with HPT persistence (33). In our cases of unilateral clearance the next day PTH decrease was significant and only one patient presented a mild biochemical persistence. The value of next day PTH in MEN1 patients deserves further research, although it is a good predictor to resolution of renal HPT (34).

One problem to be considered is that if HPT was clearly severe enough and it started in early life, would this indicate a more aggressive disease? Would it possibly lead to early recurrence if less extensive procedures were chosen? It is worth to comment that we have experience with some young patients presenting severe HPT leading to discover MEN1 followed by diagnosis of very mild HPT in their parents or grandparents. However, there is no clear evidence of more aggressive HPT in adolescents (6). Thus, the search for some additional genetic information would be extremely valuable in order to guide properly patient selection or exclusion for less aggressive operations (35). The role of mutation negativity for this purpose deserves also further research (6).

There is no consistent data on the long-term effects of hypoparathyroidism in MEN1 patients, but it is reasonable to estimate that they are at least similar to those in the general population, as cataracts, basal ganglia calcification, nephrocalcinosis, or nephrolithiasis (36).

Although we had a good experience with cryopreserved parathyroid tissue in MEN1 (37), it is not available anymore, due to logistic problems (38). Thus, the lack of an alternative to mitigate postoperative hypoparathyroidism should be included in the equation of planning the extent of the operation.

There are several limitations of the present analysis. The major one is the limited number of patients younger than 19 y. Also, the short follow up of the patients submitted to I-LSTPTX (50% of them had a very short follow up and their outcome is still unpredictable, although they are doing well until the present time) represents an additional limitation of our study.

The present study supports that HPT/MEN1 patients extensively explored during surgery attempting reach STPTX or TPTX+AG are in higher risk of develop hypoparathyroidism with similar frequency that observed with TPTX+AG. Thus, young cases (< 50 y) and/or with a potentially lower number of enlarged glands based on careful preoperative imaging could be selected to less extensive procedures as I-LSTPTX or unilateral clearance. A largest cohort of cases operated by these two later techniques may prove preliminary evidence reported in our present study. Furthermore, although this proposal is not fully supported by our limited data from the very young cases (< 20 y), this less extensive surgical protocols in adolescents could potentially avoid or postpone adverse effects of the early onset of permanent hypoparathyroidism during critical period of increased bone modeling necessary to skeletal formation. Despite a higher risk of recurrence and potential future reoperation with this approach, we postulated that by less extensive first surgery, they potentially have the additional benefit of suffer lower risk of post-surgical complications, especially if unilateral clearance was the first procedure.

Further studies with larger series of MEN1 cases and long-term follow-up may prove preliminaries evidences of the present study suggesting that I-LSTPTX or unilateral clearance is adequate in selected group of patients with HPT/MEN1 presenting lower volume of disease detected by preoperative imaging studies.

## Ethics Statement

This retrospective study was carried out in accordance with the recommendations of Brazilian Resolution 466/12 on Human Research and Institutional Review Board of Hospital das Clinicas. Privacy is fully respected and due to its retrospective nature, no informed consent was required. The study is in accordance with the Declaration of Helsinki. This study is part of a protocol was approved by the Institutional Review Board of Hospital das Clinicas.

## Author Contributions

FM, MB, and DL designed the study, collected data, created the database, and analyzed data. FM wrote the manuscript. MB, DL, and ST followed patients up, collected data, helped in writing the manuscript. FM, MB, SA, Ad'A, and GdB operated on the patients. SA, Ad'A, and GdB helped in writing. All authors contributed to manuscript revision, read, and approved the submitted version.

### Conflict of Interest Statement

The authors declare that the research was conducted in the absence of any commercial or financial relationships that could be construed as a potential conflict of interest.
